# Costing the implementation of public health interventions in resource-limited settings: a conceptual framework

**DOI:** 10.1186/s13012-020-01047-2

**Published:** 2020-09-29

**Authors:** Hojoon Sohn, Austin Tucker, Olivia Ferguson, Isabella Gomes, David Dowdy

**Affiliations:** Department of Epidemiology, Johns Hopkins Bloomberg School of Public Health, 615 N. Wolfe Street E6531, Baltimore, MD 21205 USA

**Keywords:** Implementation strategies, Costs of implementation, Economic evaluation, Decision-making, Tuberculosis

## Abstract

**Background:**

Failing to account for the resources required to successfully implement public health interventions can lead to an underestimation of costs and budget impact, optimistic cost-effectiveness estimates, and ultimately a disconnect between published evidence and public health decision-making.

**Methods:**

We developed a conceptual framework for assessing implementation costs. We illustrate the use of this framework with case studies involving interventions for tuberculosis and HIV/AIDS in resource-limited settings.

**Results:**

Costs of implementing public health interventions may be conceptualized as occurring across three phases: design, initiation, and maintenance. In the design phase, activities include developing intervention components and establishing necessary infrastructure (e.g., technology, standard operating procedures). Initiation phase activities include training, initiation of supply chains and quality assurance procedures, and installation of equipment. Implementation costs in the maintenance phase include ongoing technical support, monitoring and evaluation, and troubleshooting unexpected obstacles. Within each phase, implementation costs can be incurred at the site of delivery (“site-specific” costs) or more centrally (“above-service” or “central” costs). For interventions evaluated in the context of research studies, implementation costs should be classified as programmatic, research-related, or shared research/program costs. Purely research-related costs are often excluded from analysis of programmatic implementation.

**Conclusions:**

In evaluating public health interventions in resource-limited settings, accounting for implementation costs enables more realistic estimates of budget impact and cost-effectiveness and provides important insights into program feasibility, scale-up, and sustainability. Assessment of implementation costs should be planned prospectively and performed in a standardized manner to ensure generalizability.

Contributions to the literature

- Economic evaluations have long been used to prioritize health interventions in resource-limited settings. However, these analyses often fail to capture the resources required to successfully implement interventions and thus may greatly underestimate the actual economic costs of the intervention.
- Most existing studies in the health economics literature in the context of implementation science are retrospective or ‘ex-post’ in nature; methods and examples of evaluating these costs prospectively are limited, particularly in resource limited settings.
- Using recent examples from the literature, we highlight the importance and potential magnitude of costs associated with implementation of public health interventions in resource constrained settings.
- We also provide a conceptual framework that details the types of economic resources utilized during the design, initiation, and maintenance phases of implementation and the implications of neglecting implementation costs in evaluating the cost-effectiveness of interventions in resource-limited settings.
- Such a framework provides greater insight into the need for implementation costing practices for more accurate economic evaluation and budget impact analysis as well as insights into program feasibility, scale up and sustainability.

## Introduction

Economic evaluations are frequently used to inform prioritization of health interventions and resource allocation for public health [1]. Unfortunately, most existing economic evaluations of public health interventions have done so retrospectively [2], thereby limiting their ability to fully assess the costs of implementation, especially during the early stages of design and initiation. A growing number of studies are exploring these implementation costs, but many continue to do so in retrospective fashion, and interventions in resource-limited settings—where implementation costs may represent a disproportionate fraction of total intervention costs—are sorely under-represented [2]. As such, most published economic evaluations may have greatly underestimated the costs of public health interventions, particularly those studied in resource-limited settings. A framework for considering and prospectively collecting implementation costs could therefore greatly improve economic evaluations of public health interventions in the coming years.

To illustrate, the Avahan initiative was a complex intervention to prevent HIV transmission across six Indian states that included peer outreach, clinical services, condom distribution, safe needle exchange, and community mobilization [3]. A cost-effectiveness analysis estimated that Avahan cost $46 per disability-adjusted life year (DALY) averted [4]. However, approximately two thirds of overall costs were incurred at the “above-service” level—involving institutions above or ancillary to the provision of care (e.g., government, non-governmental organization (NGO), or multilateral institution support or infrastructure for facilitating provision of care). Had these costs of implementation not been counterbalanced by four years of service delivery, the cost per person reached could have been four times higher—with qualitatively important cost-effectiveness implications [5]. Such implementation costs are frequently neglected in economic evaluations, leading to underestimated costs, optimistic cost-effectiveness estimates, and a disconnect between published evidence and public health decision-making. This disconnect is particularly stark in settings where resources are most constrained.

Another illustration of the importance of implementation costing is the recently published PopART (HPTN 071) trial of universal testing and treatment for HIV in South Africa and Zambia [6]. This trial, conducted over 5 years, mobilized 740 community HIV care providers working in pairs (each pair covering approximately 500 households) to provide comprehensive health services—from HIV counseling and rapid testing to screening for tuberculosis (TB) and sexually transmitted infections—during annual household visits. This multifaceted intervention reduced HIV infection by 30% but required extensive programmatic coordination and administrative management, including rigorous re-training and human resource management, planning of daily household visits and periodic community engagement campaigns, HIV testing drives, and centralized coordination of study activities (e.g., obtaining regulatory approvals, provision of security, and maintenance of a stable procurement and supply chain) [7]. Costs associated with the implementation of these complex and comprehensive activities—often neglected in cost-effectiveness analyses—may make the start-up of health interventions unaffordable or infeasible. This, in turn, would result in substantial waste of resources if interventions are poorly maintained or not sustained.

To provide decision-makers with an honest appraisal of the potential costs and benefits of any health intervention, it is critical to document the full range of costs required for programmatic implementation, including the costs of intervention design and local adaptation, initiation and scale-up, and maintenance/sustainability. While consideration of implementation costs is universally relevant, these costs may be proportionally more important in settings where resources are highly constrained. Here, we use the example of peripheral diagnostic testing for TB as a case study to illustrate the importance of evaluating costs throughout the implementation process and to propose a structured method for investigating these costs.

### Peripheral diagnostic testing for TB: a case study of implementing a novel health intervention in settings of severe resource constraints

An estimated 13–18% of patients diagnosed with TB in Africa and Asia are lost to follow-up before starting treatment [8]. The current standard of care for TB diagnosis involves molecular testing requiring equipment (e.g., four-module GeneXpert® instruments) that often cannot be maintained at the point of care [9]. Novel tests—including more portable devices (e.g., GeneXpert® Edge) and simpler assays (e.g., lateral-flow detection of urine lipoarabinomannan [LF-LAM])—that can diagnose TB at the point of treatment have been prioritized as a means of reducing diagnostic delays and losses to follow-up [10]. Cost-effectiveness analyses of peripherally implemented TB diagnostic tests are emerging [11]; however, existing analyses may not fully account for the costs required to implement these novel assays in practice.

### Costing the implementation process: a framework

Similar to many consumer products, public health interventions can be conceptualized as having a “product life cycle,” with well-defined stages—design, launch, growth, maturity, and decline—from introduction to removal from the “market” [12]. Considering each of these stages is critical to the financial success of consumer products—and this is equally true for public health interventions. Although the systems required for each product life cycle phase differ, the costing of systems for launch often overlaps with those for design (preparation for launch) or growth (which starts immediately after launch)—and the decline phase acknowledges that every product (or intervention) has a finite time horizon. Thus, in mapping the structure of the product life cycle to the costing of public health interventions, one can delineate three stages: design and adaptation to the local context (“design phase” including preparation for launch), initial implementation and scale-up (“initiation phase” corresponding to launch and growth), and ongoing activities to ensure intervention sustainability (“maintenance phase” corresponding to maturity and forestalling of decline) [13].

**Fig. 1:**
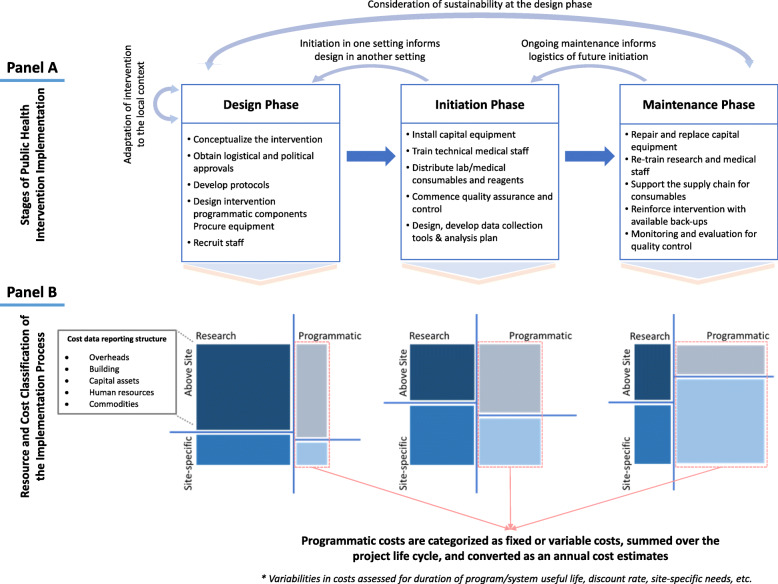
Simplified illustration of stages, activities, resource classification, and assessment of costs of public health intervention implementation process. **a** A description of the implementation process listing key components of each phase that may carry additional resource requirements is provided. Light blue arrows represent potential feedback loops between phases. **b** An illustration of resource and cost classification of the public health intervention implementation process. At each phase, resource-use for relevant activities is classified based on the location (above site vs. site-specific) of activity and degree to which activities are programmatic versus research specific. Sizes of each square (different shades of blue)—arbitrarily assigned for the purposes of demonstration—represent the corresponding size of each activity category. The increasing size of light-shaded boxes to the right indicate an increasing programmatic component as the study progresses into the maintenance phase

As illustrated in Fig. 1a, the implementation process is not necessarily unidirectional. Rather, the various phases of implementation inform one another, especially as interventions are adapted from one context to another or expanded in size or scope. For example, experiential knowledge attained during the initiation phase in one setting may inform the re-design of protocols for the design phase in a different cultural context. Similarly, the maintenance phase of an intervention in an “early adopter” country may inform policymakers and researchers about the potential sustainability of the intervention and influence considerations about design and initiation in other regions or countries. As such, it is important to individually estimate the cost of each stage of the implementation process—and to illustrate how those estimates depend on key assumptions (e.g., scale of implementation, local market rates for human resources and other costs)—so that those estimates can be generalized to other contexts where existing knowledge and infrastructure may be more or less complete.

In addition to the costs incurred at different phases of implementation, costs can be incurred at the site of delivery (“site-specific” costs; containing administrative efforts from the service delivery facility—e.g., clinic or hospital administration) or more centrally (“above-service” or “central” costs; services such as support from a district health office [DHO], NGO, or Ministry of Health [MOH]). Furthermore, health interventions are often evaluated in the context of research studies or evaluation programs, necessitating a differentiation between costs required for programmatic implementation and those incurred purely for the purposes of research or evaluation (the latter being less relevant to the cost-effectiveness of the intervention itself). Importantly, some degree of monitoring and evaluation is necessary for effective implementation and should therefore be included as a programmatic cost; however, the costs incurred by research studies and other knowledge generation activities often exceed this baseline requirement and may vary depending on the type of intervention being considered. As such, it is important to prospectively monitor and categorize costs as purely research-related (i.e., likely not to be incurred during subsequent implementation in other settings) versus necessary for programmatic implementation (even if those costs represent “research” activities) to facilitate subsequent analyses.

Table 1 provides examples of costs that may be incurred across the three phases of implementation, site-specific versus central locations, and programmatic versus research purposes—using the example of an ongoing cluster randomized trial of peripheral versus central molecular TB testing (the Xpert Performance Evaluation for Linkage to Tuberculosis Care [XPEL TB] trial) [14]. As illustrated in panel b of Fig. 1, “above-service” costs in the design and initiation phases tend to represent a greater proportion of total costs. Thus, consideration of these costs—while always important—is most critical when the costs of design and initiation are greater relative to the costs of delivery (e.g., interventions that are scaled-up to a smaller population), when interventions are being considered by decision-makers (e.g., politicians) with shorter time horizons, when interventions are expected to change in scope or cost with time (e.g., through price reductions or release of new competing interventions), or when interventions are not immediately affordable. These situations account for a large fraction of health interventions being considered for implementation in resource-limited settings. Furthermore, most public health interventions in resource-limited settings are not sustained indefinitely. Thus, rigorously measuring the costs of design and implementation and weighing those against the expected duration of the maintenance phase can improve our understanding of the costs and sustainability of public health interventions in terms of their life cycles. We now provide examples of costs that might be incurred at each phase of the implementation process (summarized in Table 2).

**Table 1:** Descriptions of key activities by study phases (XPEL study example)

Classification of activities	Research or knowledge generation costs^a^	Programmatic costs	Shared research/program costs
**Design phase**			
Central	• Ministry of Health (MoH), provincial, and district level approvals for the research study • Institutional Review Board approvals • Development of study CRFs (clinical outcomes) • Development of research database (RedCap* program) • Development of study protocols • International collaborator site visits and periodic study calls	• Central procurement of GeneXpert machines, related equipment (e.g., solar panels and external batteries), Xpert Ultra cartridges, and associated supplies • Development of SOPs* for Xpert testing, troubleshooting and QA/QC manuals, and management of testing equipment	• Recruitment of study staff • Focus group discussions
Site-specific	• Site visits for site selection • Review of clinical and laboratory data	• N/A	• Sensitization meetings at district health office and potential study sites • Pilot studies conducted at select potential study sites
**Initiation phase**			
Central	• Establish clinical and laboratory data monitoring (including National TB Reference Laboratory) • Management of regulatory processes with MoH • Development of data collection plans for health economics study • International collaborator site visits and periodic study calls	• Development of plans and organization of site visit and training • Stock management (medical and laboratory consumables and Xpert cartridges)	• Study database management (clinical and programmatic) • Data quality checks • Weekly study call (implementation issues, study data checks, etc.)
Site-specific	• Management of provincial, district level regulatory processes	• Installation of solar panel and GeneXpert equipment (including GX Alert system) • Distribution of Xpert cartridges and laboratory consumables • Training of laboratory personnel and technical support for Xpert testing	• Site sensitization meetings (w/ routine clinic staff) at both intervention and control sites • Initial participant enrolment • Establishment of data collection and follow-up plans • Interim adjustments in implementation plans (including addition or exclusion of sites) • Recruitment of study contact persons at each site (for data monitoring and study logistics purposes)
**Maintenance phase**			
Central	• Management and monitoring of study data • International collaborator site visits and periodic study calls • Data analyses and reporting	• Stock management (medical and laboratory consumables and Xpert cartridges) • Site visit organizations and communications	• Study database management (clinical and programmatic) • Central database data review and quality checks • Weekly study call (implementation issues, study data checks, etc.) • Central study team human resource management
Site-specific	• Site-specific data issue troubleshooting	• Ongoing technical support and troubleshooting (for GeneXpert and Solar panel equipment) • Distribution of Xpert cartridges and laboratory consumables • Procurement and replacement of key equipment (if broken) • QA/QC of Xpert testing • Review of GX Alert data • Periodic EQA and re-training for Xpert testing	• Quarterly site visit • Participant enrolment • Adjustments in site-specific operations • Ongoing human resource management at study sites • Data monitoring and quality checks

^a^Research or knowledge generation costs that would be required for programmatic implementation in other settings (for example, ongoing monitoring and evaluation) should be clearly delineated and considered as programmatic costs in most analyses

*SOP* Standard operation procedure, *QA* Quality assurance, *QC* Quality control, *EQA* External quality control, *GX Alert System* software system to centrally report site-specific Xpert testing statistics, equipment and testing troubleshooting, and testing operations

**Table 2:** Interventions for peripheral diagnosis of tuberculosis (TB) likely to incur large costs in each phase of implementation

Implementation phase	Application	Example
Design phase	Interventions needing large capital outlay for design and adaptation with uncertain scale-up	Implementation of mHealth-facilitated contact investigation for TB requiring procurement and tailoring of software packages
Initiation phase	Simple interventions with low consumable costs that require changes in policy and workflow	Low-cost lateral-flow LAM assay for TB that necessitates a new supply chain, clinical algorithms, and training of personnel
Maintenance phase	Interventions that require continued infrastructure support and quality control	Molecular diagnosis of TB with equipment (e.g., Xpert MTB/RIF®) that necessitates service contracts for equipment failure and external quality assurance of test results

### Design phase

Implementation costs incurred during the design and adaptation of health interventions may include crafting of appropriate policies and algorithms, obtaining political and administrative approvals, developing necessary infrastructure, engagement of relevant stakeholders, and pilot testing in the local context. Where these costs are large and must be incurred before scale-up is certain, they may represent a majority of the total cost of the intervention. For example, in a trial of mobile Health-facilitated home-based contact investigation for TB in Uganda, a software package was required to generate short messaging service (SMS) reminders to facilitate home-based screening, incurring large up-front costs for an initial service contract and adaptation to the Ugandan telecommunications infrastructure [15]. While this intervention was only delivered to 372 households (919 contacts screened), the cost of designing and developing the intervention ($137 per contact screened, of which $90 was for software development) overshadowed the cost of intervention delivery ($54 per contact screened) [16]. This initial outlay for intervention design could be partially counterbalanced by scale-up to a broader population but would probably be incurred again if the intervention were implemented in a different country with a different telecommunications system. Importantly, these costs might vary considerably across countries, regions, and facilities with different levels of underlying infrastructure—and these differences would need to be considered by both researchers (aiming to produce generalizable knowledge) and implementers (aiming to accurately estimate design costs in their unique contexts). Failure to consider the cost of designing the intervention and adapting it to the local context would have resulted in both a dramatic overestimation of cost-effectiveness and an incomplete understanding of the generalizability of cost and cost-effectiveness estimates for implementation of the intervention in other settings.

### Initiation phase

As interventions are initially rolled out and scaled-up, many types of implementation costs (e.g., training materials and personnel, infrastructure necessary to launch implementing teams) are incurred up-front. These fixed costs often do not vary with the level of service output. The contribution of up-front costs to the overall cost and cost-effectiveness of a program may vary considerably across implementation sites depending on each site’s operational and infrastructural capacity and implementation outcomes such as program reach (i.e., the number and representativeness of people engaged by the program) [17]. These costs are often not adequately considered in traditional cost-effectiveness estimates. This is particularly problematic when variable costs (e.g., medical consumables, test kits, and unit staffing costs) to deliver the intervention are low, but the intervention has high implementation and operating costs. For example, the Alere Determine^TM^ TB LAM Ag assay (LF-LAM) is a simple dipstick-based diagnostic test for TB that costs less than $2 per test kit and requires minimal operator time [18]. Cost-effectiveness analyses have therefore considered the unit cost of LF-LAM to be between $3 and $4 per test [11]. However, this estimate fails to account for the “above-service” (and service-level) costs required to implement LAM—including building costs, staff salaries, utilities, and supplies necessary for such activities as preparing clinics and training clinical staff, coordinating logistics during implementation, assuring fidelity to (often complex) policy guidance, and establishing a reliable supply chain [10]. After incorporating these costs, the unit cost of LF-LAM in South Africa was estimated at over $23 per patient tested—approximately seven-fold higher than the simple estimate based primarily on consumable costs alone [19]. Such underestimates of intervention costs (when costs of implementation and scale-up are not incorporated) are unfortunately very common in the scientific literature and lead to reported cost estimates that do not reflect programmatic realities on the ground**.**

### Maintenance phase

Although implementation costs are often proportionally lower during the maintenance phase (Fig. 1b), they should not be ignored entirely—particularly for interventions that require infrastructure and/or procedures that require ongoing upkeep or quality assurance. For example, in the XPEL trial, single-module GeneXpert devices were installed to enable point-of-treatment diagnosis at 10 peripheral health centers in Uganda. Maintaining these devices requires assurance of a stable electrical supply (e.g., installation and maintenance of solar panels), backup testing systems for when devices temporarily fail (which occurred at 6 sites over a 14-month period), security to prevent theft of computers and other electronics, service contracts to perform repairs, routine monitoring and evaluation, maintenance of a reliable supply chain of diagnostic cartridges, and external quality assurance to ensure ongoing high-quality testing by mid-level staff. Corresponding costs varied from one site to another and when considered in full, costs associated with the maintenance operations accounted for > 12% of the total unit cost of peripheral Xpert testing, even when only considering costs beyond the initiation phase (unpublished data). As the level of technology incorporated in new health interventions often outpaces the establishment of corresponding infrastructure in many global settings, explicitly estimating the costs of maintaining those interventions and ensuring their sustainability will become increasingly important [20].

### A way forward

The examples above help illustrate the importance of considering costs at each stage of the implementation process. This structured approach to costing of the implementation process suggests three priorities for improving cost-effectiveness analyses of health interventions in resource-limited settings.

First, cost-effectiveness analyses should not rely entirely on budgetary information but should explicitly consider activities and resources required for successful implementation. Examples of approaches for such implementation costing include collection of routine activity logs of implementing staff, structured discussions with field personnel to enable health economists to understand the extent of resources required for major implementation activities (e.g., trainings, site initiation), and ongoing documentation of specific challenges in implementation (e.g., sites in which implementation failed, staff leaving).

Second, implementation cost data should be classified by resource type, key activities, phase of implementation (e.g., as described in Fig. 1), site level (site-specific vs “above-service”), and as programmatic versus non-programmatic (research) so that implementation costs can be generalized to other settings and key drivers of implementation costs can be identified. In costing the implementation process, assumptions can often be made when empiric data are not immediately available—but appropriate documentation and categorization of data and assumptions are critical if generalizable knowledge is to be generated.

Third, costing activities should generally be planned prospectively before implementation begins (so the process of implementation can be costed). While retrospective estimation of implementation costs is often feasible, such estimates are often subject to recall bias and difficult to appropriately classify retroactively. Early involvement of health economists (with experience in empiric costing activities) can therefore be very useful in evaluating the costs of implementation.

One final consideration is whether detailed measurement of implementation costs is justified for a particular implementation research study. In making this assessment, investigators should consider two questions. First, if the intervention is found to be effective, is implementation likely to be influenced by considerations of cost-effectiveness and/or budget impact? Second, are there sufficient uncertainties in the costs of implementing the intervention that an empiric estimation of costs is warranted (as opposed to simply using pre-existing cost estimates from the literature)? In most cases, the answer to the first question will be yes, and the answer to the second question will be no, meaning that an empiric estimation of costs is scientifically justified. When this is the case, investigators must then evaluate whether the budget exists to measure these costs and (given limited financial resources) whether other scientific questions are more pressing.

## Conclusions

In summary, we argue that the costs of implementing health interventions in resource-limited settings are often very substantial, but generally neglected in economic evaluation. We provide a framework for effectively conceptualizing and prospectively measuring these costs, which—if incorporated appropriately—can improve the linkage between published results of cost-effectiveness analyses and the realities of implementation in the field.

## Data Availability

Not applicable
